# Role of Ethylene Biosynthesis Genes in the Regulation of Salt Stress and Drought Stress Tolerance in Petunia

**DOI:** 10.3389/fpls.2022.844449

**Published:** 2022-02-23

**Authors:** Aung Htay Naing, Jova Riza Campol, Hyunhee Kang, Junping Xu, Mi Young Chung, Chang Kil Kim

**Affiliations:** ^1^Department of Horticulture, Kyungpook National University, Daegu, South Korea; ^2^Department of Agricultural Education, Sunchon National University, Suncheon, South Korea

**Keywords:** abiotic stress, ethylene signaling, gene expression, ion homeostasis, mutant, plant growth

## Abstract

Ethylene plays a critical signaling role in the abiotic stress tolerance mechanism. However, the role of ethylene in regulating abiotic stress tolerance in petunia has not been well-investigated, and the underlying molecular mechanism by which ethylene regulates abiotic stress tolerance is still unknown. Therefore, we examined the involvement of ethylene in salt and drought stress tolerance of petunia using the petunia wild type cv. “Merage Rose” and the ethylene biosynthesis genes (*PhACO1* and *PhACO3*)-edited mutants (*phaco1* and *phaco3*). Here, we discovered that editing *Ph*ACO*1* and *PhACO3* reduced ethylene production in the mutants, and mutants were more sensitive to salt and drought stress than the wild type (WT). This was proven by the better outcomes of plant growth and physiological parameters and ion homeostasis in WT over the mutants. Molecular analysis revealed that the expression levels of the genes associated with antioxidant, proline synthesis, ABA synthesis and signaling, and ethylene signaling differed significantly between the WT and mutants, indicating the role of ethylene in the transcriptional regulation of the genes associated with abiotic stress tolerance. This study highlights the involvement of ethylene in abiotic stress adaptation and provides a physiological and molecular understanding of the role of ethylene in abiotic stress response in petunia. Furthermore, the finding alerts researchers to consider the negative effects of ethylene reduction on abiotic stress tolerance when editing the ethylene biosynthesis genes to improve the postharvest quality of horticultural crops.

## Introduction

Ethylene is considered a key regulator of plant developmental and physiological processes ranging from seed germination to senescence (Pierik et al., 2006). Additionally, it acts as a crucial signaling molecule in the abiotic stress tolerance mechanism because plants modulate ethylene to activate signaling pathways that protect them from the harmful effects of abiotic stress (Cao et al., 2007; Peng et al., 2014a; Shen et al., 2014; Zhang et al., 2016). However, a decisive conclusion on whether ethylene plays a positive role in plant response to abiotic stress could not be reached at this time. While many researchers reported a positive role of ethylene or its precursor 1-aminocyclopropane-1-carboxylate (ACC) in stress tolerance of various plant species, such as corn, *Arabidopsis*, tomato, grapevines (Lin et al., 2012; Yang et al., 2013; Freitas et al., 2017; Gharbi et al., 2017; Xu et al., 2019), other researchers claimed a negative role of ethylene in some plant growth (such as *Cucurbita pepo*, tomato, *Arabidopsis*, and tobacco) under abiotic stress (Albacete et al., 2009; Wi et al., 2010; Dong et al., 2011; Cebrián et al., 2021). The role of ethylene in the response regulation to abiotic stress depends on its level in plant tissue and plants’ sensitivity to it, as the optimal ethylene level for normal plant growth may vary at different stages and in different plant species (Khan et al., 2008; Tao et al., 2015).

Ethylene, derived from methionine, is converted into S-adenosyl-L-methionine (SAM) by SAM synthetase. SAM is then converted to 1-aminocyclopropane-1-carboxylic acid (ACC) by ACC synthetase (ACS). Finally, ACC is converted to ethylene by ACC oxidase (ACO; Yang and Hoffman, 1984; Kende, 1993). As ACO is involved in the final step of the ethylene biosynthesis pathway, *ACO* gene encoding the ACO enzyme is an ideal candidate to study ethylene production in plants; its regulatory role in ethylene production has been reported in many horticultural crops (Houben and Van de Poel, 2019). For example, petunia has been broadly used as a model in plant genetics research (Vandenbussche et al., 2016). The ethylene production in petunia floral organs has been investigated and coincided with biosynthesis-related genes *PhACO1*, *PhACO3*, and *PhACO4* expressions (Tang et al., 1993; Tang and Woodson, 1996; Huang et al., 2007). Recently, our research group also validated the association between ethylene production and gene expression in the flower of *P. hybrida* cv. “Mirage Rose” (Xu et al., 2020). Furthermore, when genes were edited using the CRISPR/Cas9 tool, there was a significant reduction in ethylene production in floral organs and prolongation of flower longevity (Xu et al., 2020, 2021), However, editing of the genes reduced ethylene production in seeds and negatively affected seed germination (Naing et al., 2021a).

As described above, ethylene can act as either a positive or negative regulator of abiotic stress tolerance, whereas the expression of the genes (*ACS* and *ACO*) involved in ethylene biosynthesis pawthway and of the genes ethylene resistance 2 (*ETR2*), ethylene response sensor 1 (*ERS1*), ethylene insensitive 2 (*EIN2*), and EIN3-like (*EIL*) involved in ethylene signaling pathway is critical in plant responses to abiotic stress (Dong et al., 2011; Peng et al., 2014a; Müller and Munnébosch, 2015; Yang et al., 2015; Gharbi et al., 2017; Riyazuddin et al., 2020). However, the expression of the genes associated with stress tolerance varied depending on the plant species; for example, *ETR1* was upregulated in cotton under both short- and longtime salt treatments (Peng et al., 2014b), while its expression was suppressed in *Arabidopsis* under salt stress (Zhao and Schaller, 2004). Although the molecular mechanisms underlying the involvement of ethylene in abiotic stress tolerance have been widely studied in other plants, it remains unknown in petunia. It is interesting to elucidate how ethylene is involved in the abiotic stress tolerance mechanisms in petunia. Therefore, we used petunia mutants with edited ethylene biosynthesis genes (*ACO1* or *ACO3*) and wild type (WT) to investigate their tolerance to salt and drought stresses, and we assessed the expressional changes of ethylene biosynthesis, receptor, and signaling genes between the before and after stress conditions.

Several plant species have revealed key physiological processes that control ion, water, and reactive oxygen species (ROS) homeostasis during abiotic stress (Munns and Tester, 2008; Miller et al., 2010; Tavakkoli et al., 2011). Overall, stomata density was reduced during abiotic stress to control transpiration rates, delay water loss, and mitigate the negative effects of osmotic stress and ion toxicity on shoot growth (Passioura and Munns, 2000; Abbruzzese et al., 2009; Hughes et al., 2017; Naing et al., 2017). Additionally, under abiotic stress, plants generated antioxidants, such as superoxide dismutase (SOD), catalase (CAT), peroxidase (POD), and proline enzymes to scavenge abiotic stress-induced ROS to protect them from oxidative stress (Müller and Munnébosch, 2015; Naing et al., 2018, 2021b). Moreover, abscisic acid (ABA) was observed to be involved in petunia drought stress resistance due to its rise in petunia leaves during drought stress (Kim et al., 2012). Similarly, the application of exogenous ABA improves drought tolerance in other plant species (Waterland et al., 2010; Du et al., 2013; Zeinali et al., 2014; Wei et al., 2015). Therefore, the changes in the expression of the genes involved in ABA synthesis and signaling pathways, including the antioxidant genes (*SOD*, *CAT*, and *POD*), proline-related gene (*Osmotin*), and the genes encoding 9-cis-epoxycarotenoid dioxygenase 1 (*NCED1*), abscisic aldehyde oxidase 31 *(AAO31)*, and phospholipase Dα *(PLDα)*, should be assessed before and after stress conditions.

In this study, we exposed wild-type (WT) petunia cv. “Mirage Rose” and two petunia mutant (*phaco1* and *phaco3*) seedlings to salt and drought stresses and investigated their tolerance to the stresses to learn how ethylene is involved in the stress tolerance mechanism by assessing the morphological and physiological parameters associated with stress tolerance. Moreover, we discovered the molecular mechanisms underlying the involvement of ethylene in stress tolerance, by analyzing the genes involved in ethylene biosynthesis, ethylene signaling, antioxidants, and ABA biosynthesis.

## Materials and Methods

### Plant Materials

In our previous works (Xu et al., 2020, 2021), we used the CRISPR/Cas9 tool to edit the ethylene biosynthesis gene *PhACO1* and *PhACO3* separately in the *Petunia hybrida* cv. “Mirage Rose,” and we obtained different homozygous *aco1* and *aco3* mutants. In this study, we investigated the role of ethylene in abiotic stress tolerance using two different homozygous lines per mutant [*aco1* (91-1 and 36-4) and *aco3* (32-15 and 14-10)]. Supplementary Table 1 shows their zygosity and genotypes. Every 400 seeds of the homozygous mutants (*aco1* and *aco3*) and WT were sown in plastic trays containing peat-based soil and the trays were placed in a greenhouse set at 25°C and ~ 60% relative humidity. Seeds were allowed to germinate for 5 weeks before selecting germinated plants (about 200 plants) with uniform sizes from each mutant and WT plant for salt and drought stress treatments.

### Salt and Drought Stress Treatments

Five-week-old homozygous mutants [*aco1* (91-1 and 36-4) and *aco3* (32-15 and 14-10)] and WT plants were transferred to plastic pots, which had holes for drainage, containing well-prepared peat-based soil with the same water content. Then, the plants were allowed to grow in the pots for 1 week, and we assessed growth parameters (plant height, plant fresh weight, and the number of leaves) in 30 plants each (ten plants per replication), but physiological parameters (SPAD value, stomata density, ion content (only for salt stress), ethylene production, and relative water content) were assessed in 15 plants each (five plants per replication) before the stress treatments. Furthermore, we sampled leaves from three different plants of the mutants and WT for RNA extraction and gene expression analyses. Following the measurement and samplings, the plants were discarded.

For salt stress treatment, the 6-week-old plants were placed in the same greenhouse and irrigated with water (50 ml each per plant) containing sodium chloride (NaCl; 50 mm). After 3 days, we watered the plants again with a concentration of 100 mm. After the next 3 days, they were subsequently watered with a final concentration of 150 mm and allowed to grow for the next 4 days.

For drought stress treatment, as done for salt stress, the 6-week-old plants were placed in the same greenhouse but the plants were placed under drought conditions without watering them for 10 days.

For each stress treatment, 20 plants were used, with three replicates for each mutant and WT. They were evaluated for the same growth and physiological parameters at the end of the salt and drought stresses. Growth parameters, such as plant height, number of leaves, and plant fresh weight were evaluated from 30 plants (ten plants per replication) of the mutants and WT. We analyzed physiological parameters, such as the relative water content (RWC), stomata density, ion content, ethylene production, and SPAD values, from 15 plants (five plants each per replication). Also, three different biological leaves were sampled from three different plants each for gene expression analysis.

### Measurement of Plant Growth Parameters

We measured plant height from the crown portion to the plant shoot tip, and only expanded leaves were counted for the number of leaves. For fresh weight, we removed the plants from the pots and thoroughly cleaned the soil attached to the roots. The plants were then immediately weighed using a portable microbalance.

### Measurement of RWC, Stomata Density, and SPAD Value

Leaf RWC and stomata density were assessed using the methods described by Naing et al. (2017). The fifth leaves from the top of the plants were used for the analyses. The number of closed and open stomata per sample was also counted. Also, we measured the SPAD value from the same position leaves using a chlorophyll meter (SPAD-502, Minolta).

### Ion Content Determination

Leaves (approximately 300 g each) were sampled from the mutants and WT plants, before and after salt stress treatment. Then, the samples were placed at dry-oven until they were completely dried. After that, we determined the presence of Na^+^, K^+^, Mg^2+^, and Ca^2+^ ion contents in each sample using an Inductively Coupled Plasma Optical Emission Spectrometer (Varian 720-ES ICP OES, Australia). The analysis was repeated thrice.

### Determination of Ethylene Production

We quantified the ethylene production in plant leaves using the Xu et al. method (Xu et al., 2020, 2021). Briefly, leaf samples (approximately 100 mg each) were collected from different plants and immediately placed in a 50 ml glass tube and sealed with a rubber septum for 24 h. After that, we measured ethylene production in each sample using gas chromatography (GC-2010; Shimadzu, Tokyo, Japan).

### Expression Analysis of Ethylene Biosynthesis Genes, Ethylene Receptors and Signaling Genes, Antioxidant- and Proline-Related Genes, and Abscisic Acid Biosynthesis and Signaling Gene

The expression levels of ethylene biosynthesis genes (*ACS1*, *ACO1*, and *ACO3*), antioxidant genes (*SOD*, *POD*, and *CAT*), proline-related gene (*Osmotin*), and ethylene receptor and signaling genes (*EIL1*, *ETR2*, *ERS1*, *and EIN2*) in plant leaves before and after exposure to salt and drought stresses were determined. Additionally, we determined the expression levels of abscisic acid biosynthesis (*NCED1* and *AAO31*) and signaling gene (*PLDα*) in plant leaves before and after exposure to drought stress. We extracted total RNA from the leaves sampled for gene expression analysis, and reverse transcription was performed as described by Naing et al. (2017). We used the Real-Time PCR system (Thermo Fisher Scientific, Waltham, MA) to measure the expression levels of the investigated genes relative to those of the tubulin gene (reference gene). Further, we calculated the relative gene expression using the quantitative comparative CT method. Supplementary Table 2 shows the primers and PCR conditions used for the investigated genes. Three different biological samples (three replicates) were used for the analysis of the mutants and WT each.

### Statistical Analysis

We used SPSS version 11.09 (IBM Corporation, Armonk, United States) to statistically analyze the data, and are presented as means (of three replicates) ± standard errors. To separate the means, we employed the least significant difference test, with the level of significance set at *p <* 0.05.

## Results

### Plant Growth Under Salt and Drought Stress

**Figure 1:**
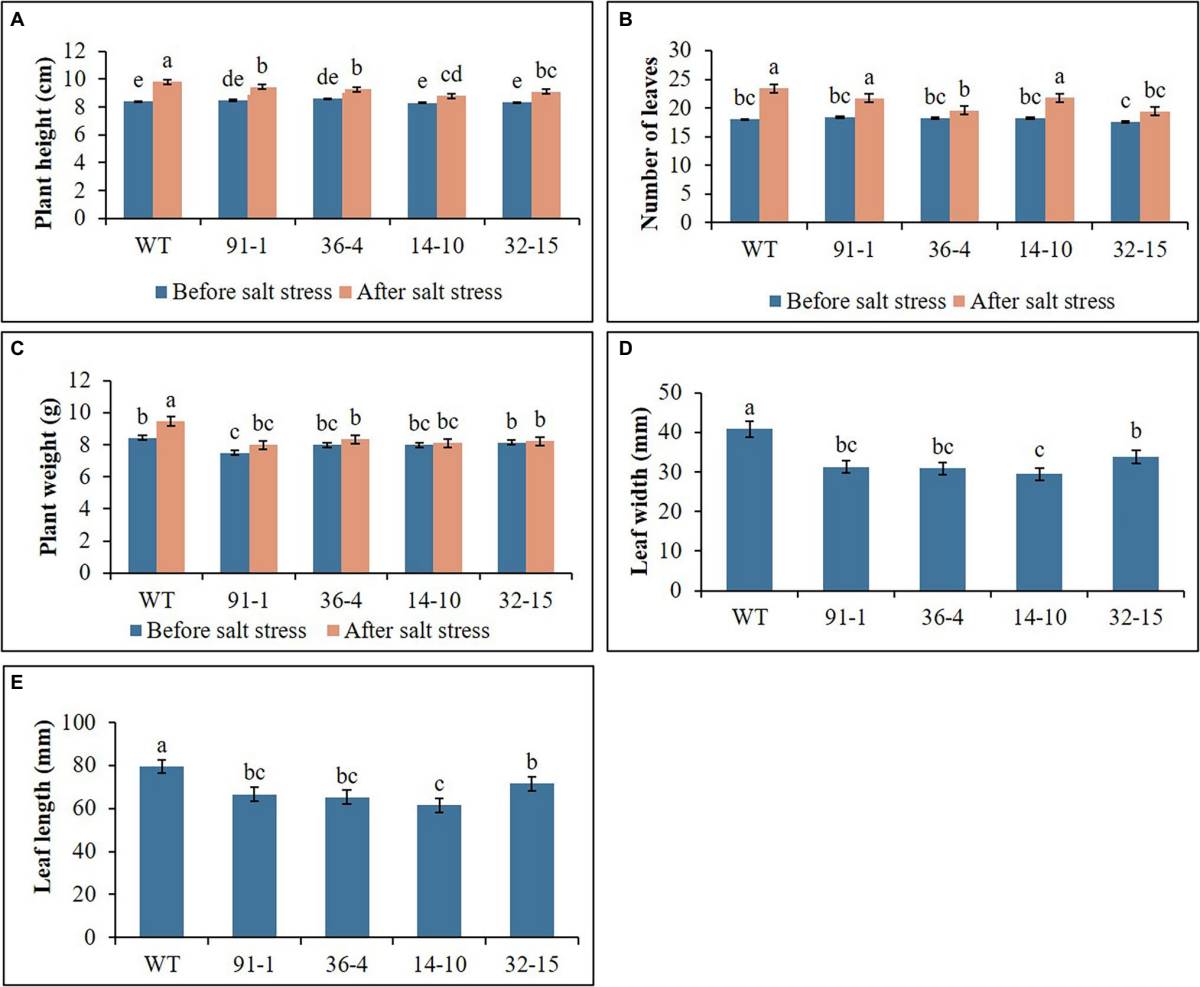
Illustration of plant growth parameters **(A–E)** of wild type (WT) petunia cv. “Mirage Rose” and mutants [*phaco1* (91-1 and 36-4) and *phaco3* (14-10 and 32-15)], before salt stress and after salt stress. Leaf size (length and width) was measured after the stress. Data represent the means of three replicates, and error bars indicate standard error. Means with the same letters are not significantly different by Least Significant Difference Test (LSDT, *p* < 0.05).

**Figure 2:**
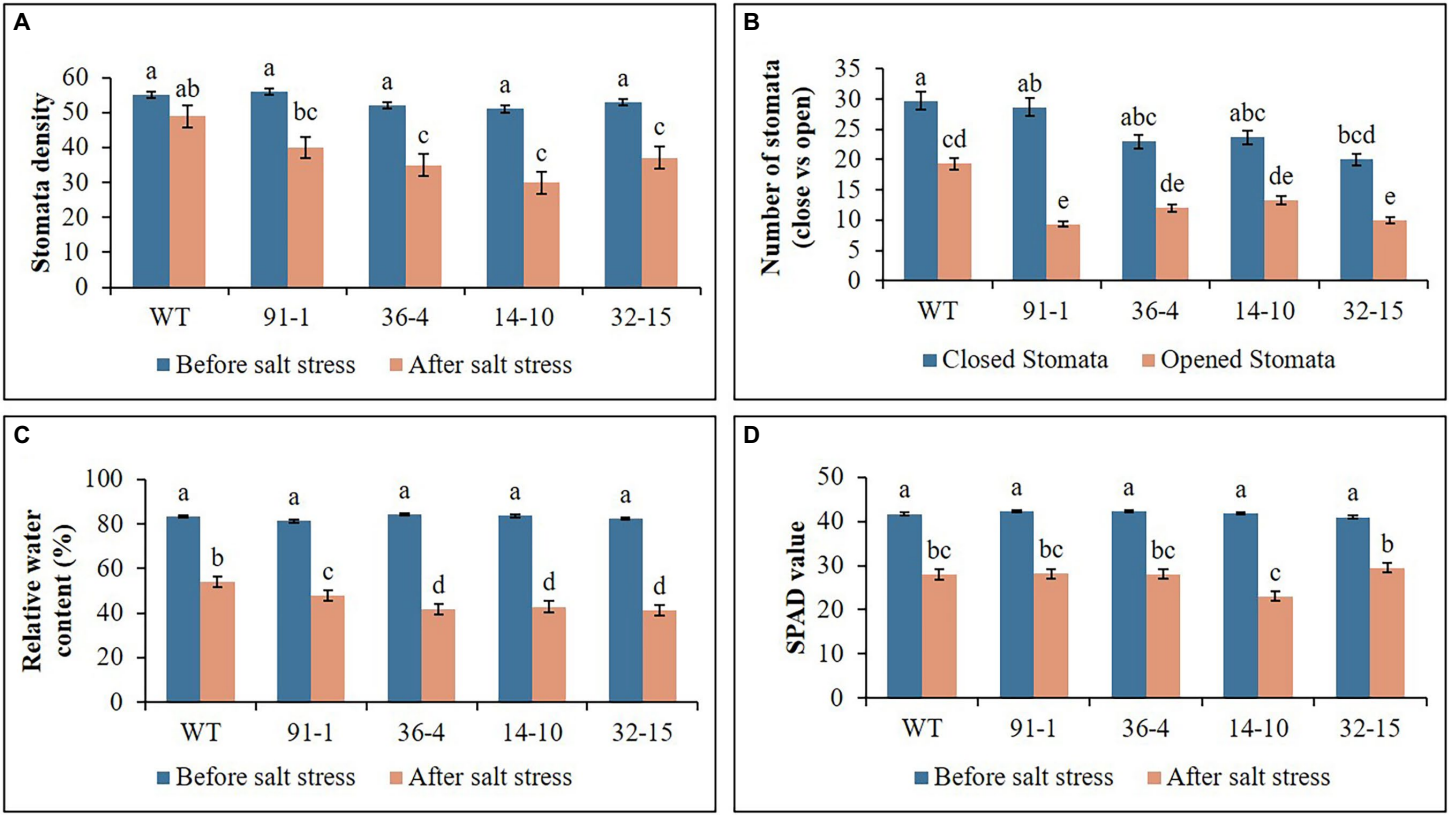
Illustration of plant physiological parameters **(A–D)** of wild type (WT) petunia cv. “Mirage Rose” and mutants [*phaco1* (91-1 and 36-4) and *phaco3* (14-10 and 32-15)], before salt stress and after salt stress. Data represent the means of three replicates, and error bars indicate standard error. Means with the same letters are not significantly different by Least Significant Difference Test (LSDT, *p* < 0.05).

**Figure 3:**
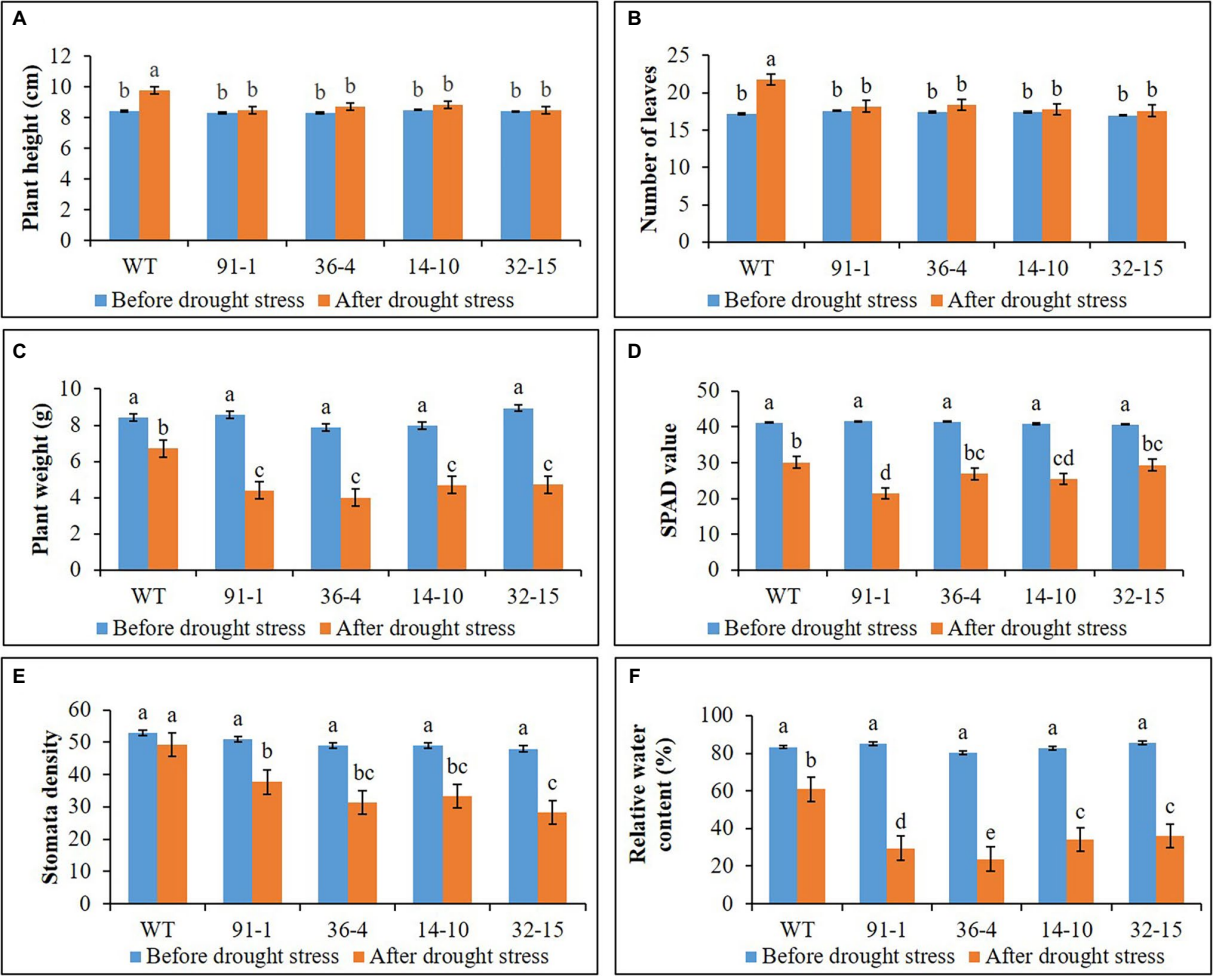
Illustration of plant growth and physiological parameters **(A–F)** of wild type (WT) petunia cv. “Mirage Rose” and mutants [*phaco1* (91-1 and 36-4) and *phaco3* (14-10 and 32-15)], before drought stress and after drought stress. Data represent the means of three replicates, and error bars indicate standard error. Means with the same letters are not significantly different by Least Significant Difference Test (LSDT, *p* < 0.05).

**Figure 4:**
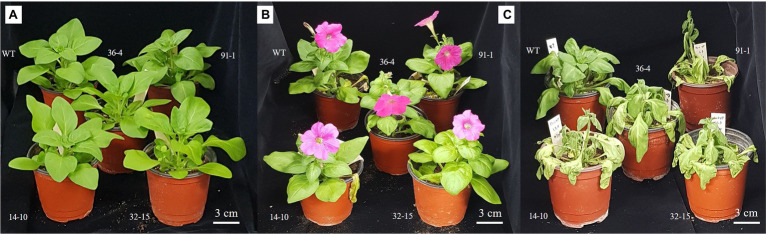
Illustration of growth status of wild type (WT) cv. “Mirage Rose” and mutants [*phaco1* (91-1 and 36-4) and *phaco3* (14-10 and 32-15)] before salt and drought stress **(A)**, end of salt stress **(B)**, and drought stress **(C)**.

The WT plants, aco1 mutants (lines; 91-1 and 36-4), and aco3 mutants (lines; 14-10 and 32-15) that exhibited similar plant growth were selected for salt and drought stress treatments. The morphological (plant height, number of leaves, and plant weight) and physiological (stomata density, RWC, and SPAD values) parameters of the selected plants before subjecting them to the stresses did not significantly differ (Figures 1–3). The growth of WT and mutant plants was not affected by salt stress treatment until they were irrigated with water containing 100 mm NaCl. However, when the NaCl concentration was increased to 150 mm, leaves curled and the internodes shortened, resulting in unhealthy-type plants compared to the stage before stress treatment (Figures 4A,B), whereas leave tissues of the mutants were discovered to be thicker than that of WT leaves. At the end of stress, WT plants exhibited a significant increase in plant growth (plant height, number of leavers, and plant fresh weight), while most mutants exhibited a slight increase in growth compared to those measured before salt stress. Overall, except for the number of leaves, all plant growth parameters observed in WT plants were significantly better than the mutants (Figure 2). These results indicated that the growth rates of mutants were slower than WT, revealing more sensitivity of mutants to salt stress than WT plants. This was associated with phenotypes of the plants, indicating that the WT plant was taller than the mutants (Figure 4B).

As observed in salt stress, WT plants exhibited a significant increase in plant height and number of leaves after 10 days of drought stress, compared to those measured before stress treatment, while such increment was not observed in all mutant plants (Figure 3). However, plant fresh weight significantly decreased in all plants subjected to drought stress for 10 days, and the reduction was more severe in mutants than in WT plants. This was supported by the phenotypes of the plants shown in Figure 4C, which revealed that practically all leaves of the mutants dropped, although some lower leaves of WT plants dropped. Therefore, these results indicate that the mutants were more sensitive to drought stress than WT plants.

### Stomata Density, RWC, and SPAD Values Under Salt and Drought Stress

**Figure 5:**
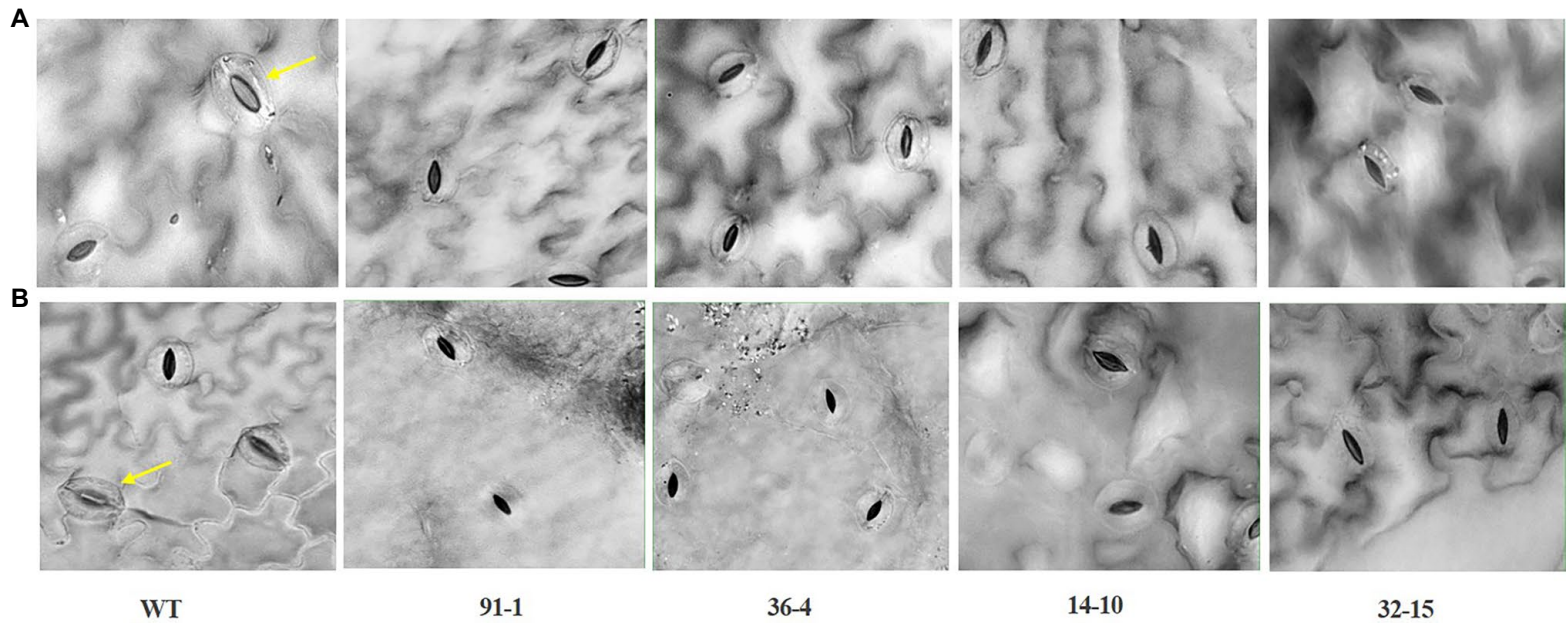
Illustration of the status of stomatal closure and opening in leaves of wild type (WT) cv. “Mirage Rose” and mutants [*phaco1* (91-1 and 36-4) and *phaco3* (14-10 and 32-15)] at the end of salt stress **(A)**, and drought stress **(B)**. Arrows indicate open stomata. Images of the stomata were taken at 40 X magnification.

The stomata density, RWC, and SPAD values, associated with plant growth against abiotic stress, were determined in the plants at the end of the salt and drought stress treatments. Significant reductions in the density, content, and SPAD values were observed in all stressed plants compared with those measured before the stress treatment, except for the stomata density of the WT plant under drought stress (Figures 2, 3). Specifically, the mutants had a greater drop in stomata density and RWC than the WT, although there was no significant difference in SPAD values between the WT and some mutants. Under drought stress, practically all stomata in the plants were closed; however, the stomata observed in the mutants were more likely closed than those of WT plants (Figure 5A). Unlike the drought stress, we observed closed and opened stomata in salt-stressed plants, with the number of open stomata higher in WT plants than the mutants (Figures 2, 5B).

### Ion Content Determination

**Figure 6:**
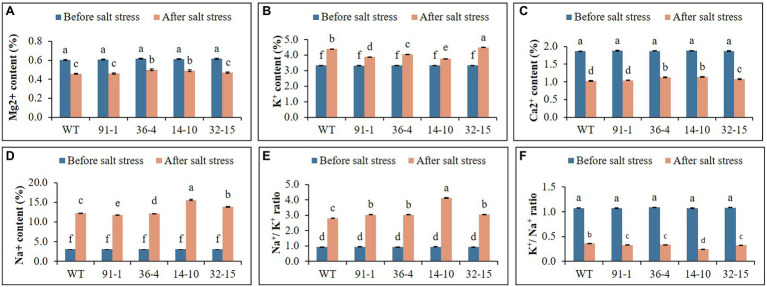
Illustration of the status of ion contents **(A–F)** accumulated in the leaves of wild type (WT) petunia cv. “Mirage Rose” and mutants [*phaco1* (91-1 and 36-4) and *phaco3* (14-10 and 32-15)], before salt stress and after salt stress. Data represent the means of three replicates, and error bars indicate standard error. Means with the same letters are not significantly different by Least Significant Difference Test (LSDT, *p* < 0.05).

Before the salt stress treatment, the ion contents (K^+^, Mg^2+^, Ca^2+^, and Na^+^) contained in the mutants and WT did not significantly differ. After exposure to salt stress, the contents of K were elevated, whereas those of Mg^2+^ and Ca^2+^ were reduced in all plants, compared to prior stress treatment. The contents of the plants exposed to salt stress differed among the mutants or between the mutants and WT. When Na^+^ content was measured in stressed plants, it was discovered to be four or five folds higher than prior stress treatment (Figure 6). After salt stress, the presence of K^+^/Na^+^ was found to be higher in WT plants than in mutants, whereas Na^+^/K^+^ content was higher in mutants than in WT.

### Expression of Antioxidant- and Proline- Related Genes Under Salt and Drought Stress

**Figure 7:**
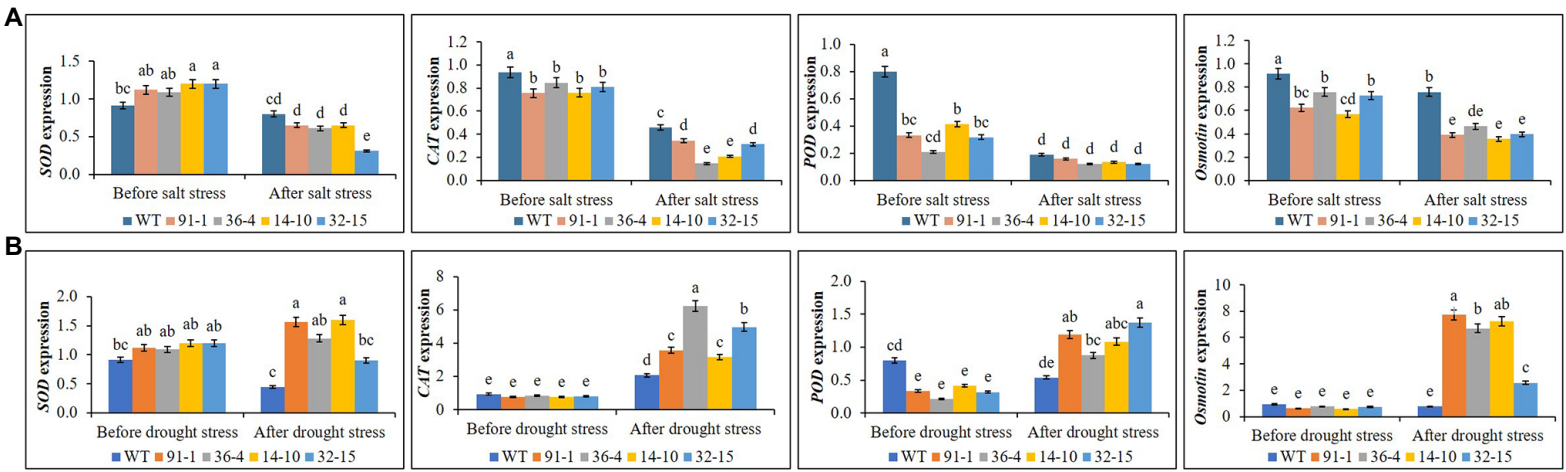
Comparison of transcript levels of antioxidant genes (*SOD, CAT*, and *POD*) and proline synthetic gene (*Osmotin*) expressed in the leaves of wild type (WT) petunia cv. “Mirage Rose” and mutants [*phaco1* (91-1 and 36-4) and *phaco3* (14-10 and 32-15)] before stress conditions and after salt stress **(A)** and drought stress conditions **(B)**. Data represent the means of three replicates, and error bars indicate standard error. Means with the same letters are not significantly different by Least Significant Different Test (LSDT, *p* < 0.05).

Before salt stress treatment, expression levels of the *CAT*, *POD*, and *Osmotin* genes were significantly higher in WT plants than in mutants, whereas those expressed in the mutants did not significantly differ from each other, except for *Osmotin* in the *aco1* mutant (36-4) and *POD* in the *aco3* mutant (14-10). When exposed to salt stress, we observed significant reductions in gene expression in all mutant and WT, but *Osmotin* expression was still significantly higher in WT plants than in mutants, and those expressed in the mutants did not significantly differ from each other under salt stress (Figure 7A), except for *SOD* in the *aco3* mutant (32–15), *CAT* in the *aco1* and *aco3* mutants (36-4 and 14-10). Unlike the salt stress, expression levels of the genes did not significantly differ between the mutants and WT or among the mutants before drought stress, except for *POD* in WT. After the drought stress treatment, we observed a drought-induced significant elevation of *CAT*, *POD*, and *Osmotin* in the mutants, despite some variation of the gene expression among the mutants (Figure 7B). Interestingly, except for *CAT*, the expression of other genes in WT plants was even slightly reduced. Under both conditions, we observed no significant variation of *SOD* in the mutants or between the mutants and WT.

### Expression of ABA Biosynthesis and Signaling Genes Under Drought Stress

**Figure 8:**
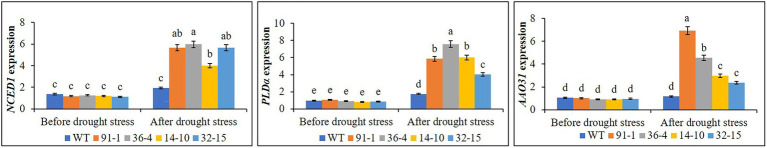
Comparison of transcript levels of ABA synthesis genes (*NCED1* and *AAO31*) and ABA signaling gene (*PLDα*) expressed in the leaves of wild type (WT) petunia cv. “Mirage Rose” and mutants [*phaco1* (91-1 and 36-4) and *phaco3* (14-10 and 32-15)] before stress conditions and after drought stress conditions. Data represent the means of three replicates, and error bars indicate standard error. Means with the same letters are not significantly different by Least Significant Different Test (LSDT, *p* < 0.05).

Before the drought stress, the expression of *NCED1*, *AAO31*, and *PLDα* did not significantly differ between the WT and mutants. However, after the drought stress, we observed drought-induced significant upregulation of genes in all mutants, but not in WT plants, except *PLDα* (Figure 8). Therefore, the expression of the genes in the mutants was significantly higher than in the WT plants after stress, despite the variation of the gene expression among the mutants.

### Ethylene Production and Expression of Ethylene Biosynthesis Genes in the Mutants and WT Plants Under Salt and Drought Stress

**Figure 9:**
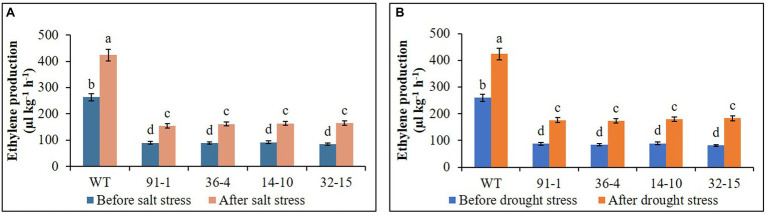
Comparison of ethylene production in the wild type (WT) petunia cv. “Mirage Rose” and mutants [*phaco1* (91-1 and 36-4) and *phaco3* (14-10 and 32-15)] before stress conditions and after salt stress **(A)** and drought stress conditions **(B)**. Data represent the means of three replicates, and error bars indicate standard error. Means with the same letters are not significantly different by Least Significant Different Test (LSDT, *p* < 0.05).

**Figure 10:**
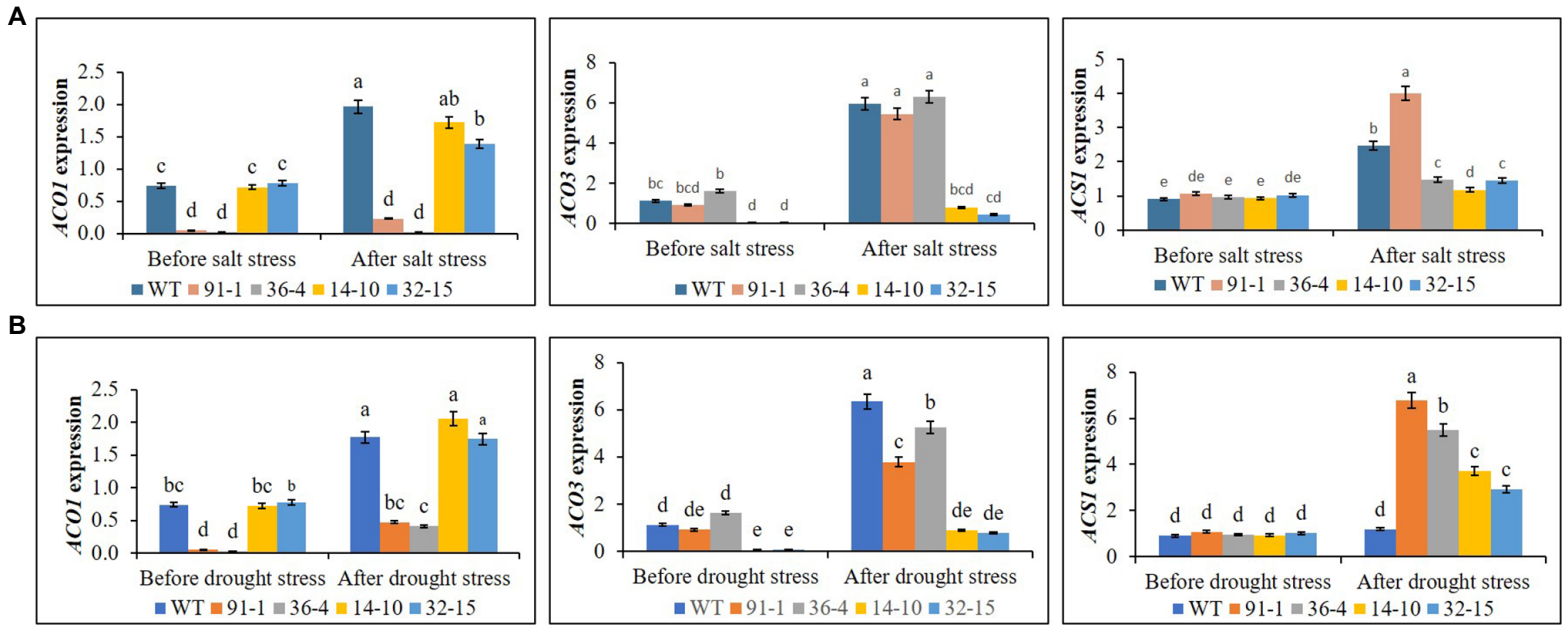
Comparison of transcript levels of ethylene biosynthesis genes (*ACS1, ACO1*, and *ACO3*) expressed in the leaves of wild type (WT) petunia cv. “Mirage Rose” and mutants [*phaco1* (91-1 and 36-4) and *phaco3* (14-10 and 32-15)] before stress condition and after salt stress **(A)** and drought stress condition **(B)**. Data represent the means of three replicates, and error bars indicate standard error. Means with the same letters are not significantly different by Least Significant Different Test (LSDT, *p* < 0.05).

Ethylene production was measured in all plants before exposing them to any stress treatment, whereas that produced in WT plant was significantly higher than all mutants, but there was no significant production of ethylene among the mutants. After exposing them to salt and drought stresses, we observed significant increases in ethylene production in all plants under both stress conditions, compared to those before stress conditions. However, those observed in WT plants were significantly higher than those in mutant plants under both stress conditions (Figure 9). When determining the expression levels of *ACS1* in all mutants and WT plants before they were exposed to salt and drought stresses, their expression levels were not significantly different from each other, in addition, those of *ACO1* in the *aco3* mutants and WT or of *ACO3* in the *aco1* mutants and WT were not also significantly different. However, *ACO1* expression in the *aco1* mutants or *ACO3* expression in the *aco3* mutants were barely detectable. After stress treatments, salt-induced significant elevations of *ACS1*, *ACO1*, and *ACO3* were, respectively, observed in WT, but, under drought stress, only *ACO1* and *ACO3* were significantly elevated in WT plants, and significant elevation of *ACS1* was not observed, compared to before stress conditions (Figure 10A). In all mutants, we detected significant induction of *ACS1* expression by both stresses, and *ACO1* expression in the *aco3* mutants, and *ACO3* expression in the *aco1* mutants, were also significantly upregulated. However, the induction of *ACO1* expression in the *aco1* mutants by salt stress or of *ACO3* expression in the *aco3* mutants by both stresses was not significantly higher than those observed before stress treatment. Under drought stress, *ACO1* expression in the *aco1* mutants was significantly upregulated but still relatively lower than those expressed in WT and *aco3* mutants (Figure 10B). These results indicate that ethylene production was associated with the expression of ethylene biosynthesis genes and that editing either *ACO1* or *ACO3* significantly inhibited ethylene production in the mutants under both normal and stress conditions, compared to WT plants.

### Expression of Ethylene Receptor and Signaling Genes Under Salt and Drought Stress

**Figure 11:**
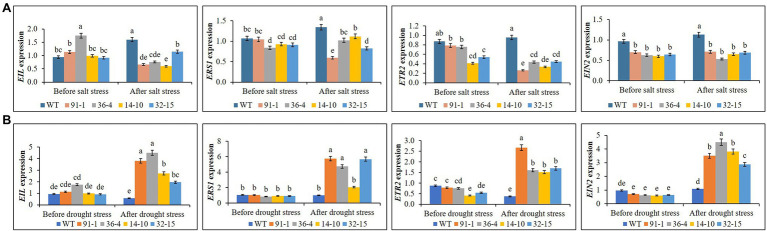
Comparison of transcript levels of ethylene receptor and signaling genes expressed in the leaves of wild type (WT) petunia cv. “Mirage Rose” and mutants [*phaco1* (91-1 and 36-4) and *phaco3* (14-10 and 32-15)] before stress condition and after salt stress **(A)** and drought stress condition **(B)**. Data represent the means of three replicates, and error bars indicate standard error. Means with the same letters are not significantly different by Least Significant Different Test (LSDT, *p* < 0.05).

We observed the differential expression of *ETR2*, *EIN2*, *EIL1*, and *ERS1* in the mutants and WT plants before exposure to salt stress. However, their expression levels did not significantly differ between the WT and the mutants or among the mutants, except in some cases. After the stress treatment, we discovered the expression of the genes in the mutants and WT plants to be slightly or significantly varied, whereas salt-mediated expression levels differed depending on the types of genes or genotypes of mutants. For example; salt suppressed *EIL1* expression in all mutants except the *aco3* mutant (14-10) and their expression levels did not significantly differ from each other, whereas we observed slightly or significantly increased *EIL1* expression in the mutant (14-10) or WT plants, compared to before stress treatments. Similarly, *ERS1* expression was suppressed in the mutant (91-1), and its elevation was observed in the mutants (36-4 and 14-10) and WT plants (Figure 11A). Moreover, we observed similar *ETR2* suppression in the mutants (91-1 and 36-4), while such significant suppression was not observed in other mutants. But, significantly suppression or elevation of *ETR2* was not observed in WT. Unlike other genes, there are no significant changes in *EIN2* expression in all mutant and WT plants after stress treatments. In contrast to the salt stress, expression levels of the detected genes did not differ significantly in all mutants and WT plants before exposure to the drought stress. However, after the drought stress, we observed drought-induced significant elevations of the genes in all mutants except for the *ERS1* gene in the *aco3* mutant (14-10), compared to before stress conditions (Figure 11B). Surprisingly, a significant elevation of the genes was not observed in WT plants under drought stress. Overall, involvement of the receptor and signaling genes in the stress tolerance was observed, however, the mode of their involvement in salt stress and drought stress differed.

## Discussion

The involvement of ethylene in the abiotic stress tolerance mechanism has been reported in many plant species, however, the role of its involvement in the mechanism remains unclear because it can act as a positive or negative regulator of the stress tolerance depending on its concentrations, plant species, and plant growth stages (Albacete et al., 2009; Wi et al., 2010; Dong et al., 2011; Freitas et al., 2017; Xu et al., 2019; Cebrián et al., 2021). In this study, we examined how ethylene affects salt and drought stress tolerance in *Petunia hybrida* cv. Mirage Rose using the ethylene biosynthesis genes (*PhACO1* and *PhACO3*) mutants (*phaco1* and *phaco3*) and WT plants.

There were no significant changes in plant growth and physiological parameters between the mutants (*phaco1*; 91-1 and 36-4, *phaco3*; 14-10 and 32-15) and WT or among the mutants before they were exposed to any stress. However, ethylene levels produced in the leaves of mutants were significantly lower than those of WT, despite no significant variation in ethylene production among the mutants. When they were exposed to salt and drought stresses, the differential response of the plants to the stresses was distinctly observed. Under salt stress, we observed a reduction of plant growth, RWC and SPAD values, and stomata density, and this could be due to the more Na^+^ content accumulation (four or five folds) in the stressed plants than before stress conditions. Because the high Na^+^ content will cause osmotic stress to the plants and might disrupt the water uptake and photosynthesis efficiency, resulting in decreased plant growth. Furthermore, the plants reduce stomata density and/or close stomata to delay water loss for sustained growth against stress. In this study, the elevation of K^+^ content was also observed in all plants under salt stress. It seemed that the plants triggered K^+^ to compete with Na^+^ in membrane transport and enzymatic functions to reduce the harmful effects of Na^+^ (Munns and Tester, 2008; Shabala, 2013; Deinlein et al., 2014; Sun et al., 2015). The importance of K^+^/Na^+^ content in salt stress tolerance has been reported in many plant species (Nadeem et al., 2006; Zhang et al., 2008; Karlidag et al., 2013; Han et al., 2014; Singh and Jha, 2016). However, the imbalance of K^+^/Na^+^ content was observed in the plants, whereas increased K^+^/Na^+^ content in WT plants over mutants would lessen the harmful effect of Na^+^ content in the former more than the latter. This was associated with the results of higher plant height, fresh weight, RWC, SPAD value, number of open stomata in the WT than the mutants. Abiotic stress often increases ROS generation in different organelles (Naing and Kim, 2021). Enzymatic antioxidants, such as SOD, CAT, and POD, are involved in the defense mechanism against the toxic effects of excess ROS; specifically, SOD removes $O_{2^{-}}$ by catalyzing its dismutation, while CAT and POD remove H_2_O_2_ (Gill and Tuteja, 2010). Similar to these enzymes, proline is also involved in the defense mechanism and is effective in scavenging OH^−^ (Smirnoff and Cumbes, 1989). Meanwhile, Osmotin induces proline biosynthesis by activating Δ^1^-pryrroline-5-carboxylate synthetase (P5GS), which catalyzes the rate-limiting step in proline biosynthesis (Delauney and Verma, 1993; Kavi Kishor et al., 1995). In this study, the suppression of *SOD*, *CAT*, *POD*, and *Osmotin* were observed in all plants compared to before stress conditions. The plants most likely consumed antioxidants and prolines to scavenge the salt-induced ROS, whereas the expression level of *Osmotin* was still significantly higher in the WT than the mutants, which might contribute to greater stress tolerance of the WT over the mutants. Previous studies using exogenous proline and transgenic plants with increased proline content revealed the importance of prolines in salt stress tolerance (Khedr et al., 2003; Simon-Sarkadi et al., 2006).

Similar to the salt stress, the reduction of the plant growth, RWC, stomata density, and SPAD values were also observed in the plants under drought stress. Additionally, all stomata were closed under drought stress. Because the plants were not watered for about 10 days, they cannot sufficiently uptake water and their RWC was significantly declined. This made the plants reduce the stomata density and close the stomata to maintain RWC and control the high transpiration rate. Moreover, a reduction in chlorophyll content or SPAD value is also one of the major responses to early drought stress. Such reduction in plant growth and disruption of the physiological process by abiotic stress has been observed in petunia (Arun et al., 2016; Ai et al., 2018; Naing et al., 2021b). In this study, the WT plant was discovered to tolerate drought stress more than the mutants. This was associated with the existence of greater plant growth and physiological parameters in WT over the mutants. Unlike the salt stress, we observed an upregulation of *CAT*, *POD*, and *Osmotin* genes in the mutants, but this was not observed in WT plants. It was likely that as the mutants were highly sensitive to drought stress, they strongly triggered antioxidant and proline-related genes to scavenge the drought-induced ROS for their survival. Whereas, WT plants that were less sensitive to the drought stress compared to the mutants might utilize existing antioxidants and prolines to protect them from the stress without further upregulating the genes. Because no significant changes in *SOD* expression were identified in the mutants or WT, it appeared that *SOD* is not involved in petunia drought tolerance. Abscisic acid (ABA) is the primary chemical signal for drought sensing. Its increase in concentration under drought stress regulates stomatal closure to prevent water loss (Zhang et al., 2004; Mishra et al., 2006). *NCED* and *AAO* are key regulatory genes of ABA biosynthesis (Seo et al., 2000; Thompson et al., 2000; Harb et al., 2010), and *PLDα* affects ABA signaling by mediating the effects of ABA on stomatal closing and opening. (Zhang et al., 2004; Mishra et al., 2006). Kim et al. (2012) discovered that petunia induced ABA under drought stress and improved tolerance to the stress. Similarly, other studies also reported an improvement of drought tolerance by applying exogenous ABA in other plant species (Waterland et al., 2010; Du et al., 2013; Zeinali et al., 2014; Wei et al., 2015). Harb et al. (2010) also reported that *PLDα* was upregulated as an early response in *Arabidopsis* leaves under moderate drought stress. In this study, we observed drought-induced upregulation of *NCED1*, *AAO31*, and *PLDα* in the mutants but not in the WT plants except *PLDα*. In a study by Kim et al. (2012), there were no significant changes in *NCED1* and *AAO31* expression in petunia under drought stress, despite the increase in leaf ABA concentration. In addition, they indicate that *de novo* ABA synthesis may not be a significant contributor to the increase in ABA concentrations in petunia leaves during drought. Therefore, although the upregulation of the *NCED1* and *AAO31* genes were not observed in the WT of this study, as observed by Kim et al. (2012), ABA concentration may increase in the WT leaves under drought stress. However, higher expression of the genes in the mutants than the WT could be explained that as the mutants were extremely sensitive to drought stress, they highly induced the ABA *via* upregulation of the genes to mediate the severity of the stress. In contrast to *NCED1* and *AAO31*, *PLDα* was significantly upregulated in the WT, which was also similar to the finding of Kim et al. (2012), who suggested that *PLDα* expression promoted stomatal closing because phosphatidic acid generated through *PLDα* activity binds to ABI1/PP2C, a key component of ABA signaling, involved in stomatal regulation (Mishra et al., 2006). We also agreed with their suggestion because, in our study, higher *PLDα* expression in the mutants than in the WT was associated with more closing of the stomata in the mutants than in the WT.

Under the stresses, the induction of ethylene in WT was approximately two- or three-fold higher than the mutants. Furthermore, we observed the stress-induced *ACS1* expression in all plants except WT under drought stress. However, the stresses did not significantly upregulate the altered *ACO1* or *ACO3* genes in their respective mutants, but they were all increased in the WT. Salt-induced *ACS* and *ACO* expression was observed in tobacco and cotton (Cao et al., 2007; Shen et al., 2014; Peng et al., 2014b; Xu et al., 2019), and the authors suggested that the promotion of ethylene production is necessary for plant adaptation to the salt stress condition. Similarly, Arraes et al. (2015) also reported drought-induced upregulation of *ACS* and *ACO* expression in soybean. Naing et al. (2021b) recently discovered drought-induced *ACO1* expression and ethylene production in the petunia. In this study, greater stress tolerance of the WT over the mutants also indicated that ethylene was necessary for the modulation of stress response and adaptation in the petunia, and it seemed that editing of the *PhACO1* or *PhACO3* suppressed the ethylene production lower than the threshold level that is required for stress adaptation, thereby making the mutants more sensitive to the stresses, compared to WT. This finding was consistent with those of previous studies revealing the positive role of ethylene in abiotic stress tolerance in other plant species (Lin et al., 2012; Yang et al., 2013; Freitas et al., 2017; Gharbi et al., 2017; Xu et al., 2019). Jiang et al. (2013) reported that ethylene overproduction in the eto1 mutant results in salinity tolerance due to improved Na^+^/K^+^ homeostasis through an RBOHF-dependent regulation of Na^+^ accumulation. de Zélicourt et al. (2018) also discovered that *Enterobacter* sp. SA187 induces salt stress tolerance in Arabidopsis through the production of KMBA that activates the ethylene pathway, and Na^+^/K^+^ content observed in the plant inoculated with SA187 was lower than that of noninoculated plants. Therefore, in this study, the existence of a higher Na^+^/K^+^ or lower K^+^/Na^+^ content in the mutants over the WT could be due to the lower ethylene production in the mutants caused by editing of the *ACO1* or *ACO3*. Moreover, previous studies also reported that ethylene can regulate stomatal closure (Shi et al., 2015) and activate ABA synthesis through the transcription of *NCED1* (Rakitin et al., 2009; Sun et al., 2010), this would help the WT to more modulate the severity of the stresses than the mutants.

Ethylene signaling is necessary for plant responses and adaptation to abiotic stress, whereas the expressions of *ETR2*, *ERS1*, *EIN2*, and *EIL*, which are involved in the ethylene signaling pathway, are critical for plant responses to abiotic stress (Cao et al., 2007; Lei et al., 2011; Peng et al., 2014a,b; Arraes et al., 2015; Ge et al., 2015; de Zélicourt et al., 2018). Peng et al. (2014a) reported that salt-induced *EIL1* confers salinity tolerance by inhibiting ROS accumulation in *Arabidopsis*, and loss-of-function mutant *ein3-1* and the double mutant *ein3eil1* were extremely sensitive to salinity, In addition, in Arabidopsis, loss-of-function of *EIN2* became more sensitive to salinity, and overexpression of the C-terminus of *EIN2* in *ein2-5* suppressed the salinity sensitivity (Cao et al., 2007; Lei et al., 2011; Peng et al., 2014a). Moreover, we observed salt-induced *ETR2* upregulation in cotton (Peng et al., 2014b). In guard cells of *Arabidopsis* leaves, *ETR1* and *ERS1* mediate both ethylene and H_2_O_2_ signaling, highlighting ethylene-mediated regulation of H_2_O_2_ concentrations during salinity stress (Ge et al., 2015). Similarly, de Zélicourt et al. (2018) also claimed that blocking the ethylene receptors by AgNO3 decreased the plants’ tolerance to salt stress. In this study, we observed transcriptional changes of the receptor and signaling genes in the WT and mutants between the before stress and after stress conditions, indicating the necessity of ethylene signaling in petunia stress adaptation. However, as observed in the aforementioned studies, salt-induced significant elevation of ethylene signaling genes (*EIL1* and *EIN2*) and receptor gene (*ERS1* and *ETR2*) were observed in WT, but not in the mutants except *ERS1* in some mutants. This supports greater WT tolerance to the salt stress over the mutant. Moreover, other studies also hypothesized the involvement of ethylene signaling in abiotic stress tolerance (Splivallo et al., 2009; Garnica-Vergara et al., 2016; Verbon and Liberman, 2016). Under drought stress, in contrast to salt stress, the genes were upregulated in the mutants that were highly sensitive to the drought, but not in the WT that was moderately sensitive to the drought. It showed that the expression patterns of the ethylene signaling genes were opposing between the salt and drought stresses, indicating that regulation of ethylene signaling in response to abiotic stress tolerance varied depending on the types of abiotic stress. Arraes et al. (2015) reported that *ETR* expression was significantly higher in the leaves of drought-sensitive soybean cultivars than drought-tolerant soybean cultivars when they were exposed to the drought stress for 150 min. However, it is still unknown the reason why the transcriptional changes of the genes were not observed in the WT between the before stress and after stress conditions, even though they were moderately sensitive to the drought. Other hormonal signaling pathways, including the ABA signaling pathway, may be involved in drought stress adaptation, whether ethylene-dependent or -independent because the ABA signaling gene *PLDα* that regulates stomatal closure was significantly upregulated in the WT. In addition, we observed ethylene-induced ABA synthesis in grapevine (Rakitin et al., 2009; Sun et al., 2010). To the best of our best knowledge, there has been no report revealing the involvement of ethylene signaling in petunia under abiotic stress. Therefore, further research would be needed to study deep insight as to how ethylene signaling is involved in petunia stress adaptation. Overall, these results indicated how ethylene is involved in the abiotic stress tolerance mechanism of petunia and alerted the researchers to consider this in the future when editing the ethylene biosynthesis genes using CRISPR/Cas9 for improvement of flower longevity and quality. The occurrence of the slight variation in plant growth, physiological parameters, and gene expression among the mutants could be attributed to the differences in edited genes and/or their deletion patterns (genotypes).

## Conclusion

We discovered that editing *PhACO1* or *PhACO3* in *P. hybrida* cv. “Mirage Rose” reduced ethylene production in the leaves of the *phaco1* and *phaco3* mutants and reduced tolerance to salt and drought stress, compared to the WT. This was proven by analyzing the plant growth and physiological parameters, indicating superior plant growth and physiological parameters in the WT to the mutants. Furthermore, we revealed the molecular mechanism by which ethylene is involved in stress tolerance by analyzing the expression levels of antioxidant and proline-related genes, ABA synthesis and signaling genes, and ethylene receptor and signaling genes in the mutants and WT plants for both before and after stress conditions. We observed that the transcriptional regulation of genes in response to the stresses varied between the mutants and WT plants. These results indicate the necessity of ethylene for abiotic stress adaptation in petunia to some extent and provide a better physiological and molecular understanding of the role of ethylene in the abiotic stress response in petunia. Additionally, the finding alerts that when editing the ethylene biosynthesis genes for the prolongation of postharvest fruit, vegetable, and flower quality, careful consideration should be taken to avoid the negative effects of ethylene reduction toward reduced tolerance to the abiotic stresses.

## Data Availability Statement

The datasets presented in this study can be found in online repositories. The names of the repository/repositories and accession number(s) can be found in the article/**Supplementary Material**.

## Author Contributions

AN designed the study, wrote, and revised the manuscript. AN and JC conducted the experiments. HK did gene expression. JX and MC assisted the experiment. CK supervised the project. All authors read and approved the final manuscript.

## Funding

This work was carried out with the support of “Cooperative Research Program for Agriculture Science & Technology Development (Project No. PJ01485801)” Rural Development Administration, Republic of Korea.

## Conflict of Interest

The authors declare that the research was conducted in the absence of any commercial or financial relationships that could be construed as a potential conflict of interest.

## Publisher’s Note

All claims expressed in this article are solely those of the authors and do not necessarily represent those of their affiliated organizations, or those of the publisher, the editors and the reviewers. Any product that may be evaluated in this article, or claim that may be made by its manufacturer, is not guaranteed or endorsed by the publisher.

## Supplementary Material

The Supplementary Material for this article can be found online at: https://www.frontiersin.org/articles/10.3389/fpls.2022.844449/full#supplementary-material
