# Supplementary material for: Role of Ethylene Biosynthesis Genes in the Regulation of Salt Stress and Drought Stress Tolerance in Petunia
Source: Front Plant Sci. 2022 Feb 23;13:844449. doi: 10.3389/fpls.2022.844449 (PMC8906779; doi:10.3389/fpls.2022.844449)
Supplement: Supplementary file 1 [file Table_1.DOCX]

Supplementary Table 1 Illustration of homozygous petunia mutants editing different *ACO* genes (*ACO1* and *ACO3*) along with their genotypes / indel pattern

| **Homozygous mutants** | **Edited gene** | **Genotype / Indel pattern** |
| --- | --- | --- |
| WT | ⅹ | ⅹ |
| 91-1 | *ACO1* | -10 Del |
| 36-4 | *ACO1* | -5 Del |
| 14-10 | *ACO3* | -360 Del |
| 32-15 | *ACO3* | +1bp A Ins |

Del#, number of base pairs deleted at the target sites; Ins#, number of bases inserted at target sites

Supplementary Table 2. Primers used for gene expression analysis using quantitative real-time PCR.

| Gene  (Accession No.) | | | Primers sequences | |
| --- | --- | --- | --- | --- |
|  |  |  | Forward primer **(5’ to 3’)** | Reverse primer **(5’ to 3’)** |
| *ACO1* | L21976.2 | | 5'- ATCAGCTTGGACAAAGTGAATGG-3' | 5'-CACCAACTCAAAGAAGCCCC-3' |
| *ACO3* | L21978.1 | | 5'-GGGGCTTCTTTGAGTTGGTGA-3' | 5'-AGATGTTAGAAACAGGAAGATGGC-3' |
| *POX* | D11396.1 | | 5'- ACTGCTCCGTCACCCAAAAC-3' | 5'- GCCCTGGTTGCTTAAGTC-3' |
| *SOD* | X14352.1 | | 5'- ACTCAGTCGTTGGAAGAGCG-3' | 5'- TGGTAAGGCTGAGTTCGTGG-3' |
| *CAT* | AY726007.1 | | 5'- CAGCCAGTGGGACGATTAGT-3' | 5'- GGCACCACAATAGAAGGGCA-3' |
| *Osmotin* | AF376058.1 | | 5'- CTTTCGCCCCAACTAAGCCT-3' | 5'- TGCACCAGGACATTCACCAT-3' |
| *ACS1* | Z18953.1 | | 5'-ACCGAGTAGTTATGGCCGGT-3' | 5'-GAATGCATCGCCAGCATCAG-3' |
| *EIL* | AY353248.1 | | 5'-CACCTCAACGGAAGTACCCG-3' | 5'-TATATGGCGGTTTCTGGCCC-3' |
| *ERS1* | DQ154118.1 | | 5'-TGGGTCCTCATGCAGTTTGC-3' | 5'-CATAACCACAGCGACCGTCT-3' |
| *ETR2* | DQ154119.1 | | 5'-GAGATAGTCAGGGTCGTGGC-3' | 5'-AGCTCGATTTTGCTCCTCCA-3' |
| *EIN2* | AY353249.1 | | 5'-ACTCATGCAAAGTCGGCCAG-3' | 5'-TGACTTGTTGCAGAACGGGG-3' |
| *NCED1* | Z97215.1 | | 5'-TGGAGGCTACTGTTGAGCTTCCTT-3' | 5'-ACCTACAAGCTTCAAGCTTGGTTT-3' |
| *AAO31* | NM_001084497.1 | | 5'-ACCACTACTTCTGGCAGCTTCAGT-3' | 5'-AATCCAGGCCACAGAGTGTCTTCA-3' |
| *PLDα* | AF201661.1 | 5'-CCTTTATCCAGAGAGCTTGGAATGC-3' | | 5'-TTTCTGCTCCAGGTAGTTCAGTCACC-3' |
| *Tublin* | SGN-U207876 | | 5'-TGGAAACTCAACCTCCATCCA-3' | 5'-TTTCGTCCATTCCTTCACCTG-3' |

*TUB: tubulin gene

PCR condition - 95℃(10min) followed by 40 cycles of [95℃(15s)-59℃(1min)] -95℃(15s)-60℃(1min)-95℃(15s)
